# A review of bioanalytical techniques for evaluation of cannabis (Marijuana, weed, Hashish) in human hair

**DOI:** 10.1186/s13065-019-0627-2

**Published:** 2019-08-14

**Authors:** Iltaf Shah, Bayan Al-Dabbagh, Alaa Eldin Salem, Saber A.A. Hamid, Neak Muhammad, Declan P. Naughton

**Affiliations:** 1 Department of Chemistry, College of Science, UAEU, Al Ain, Abu Dhabi, UAE; 2 School of Life Sciences, Pharmacy and Chemistry, Kingston University, Surrey, UK

**Keywords:** Hair analysis, Psychoactive drugs, Bioanalytical techniques, GC–MS, DART-MS, LC–MS/MS, Cannabis, Marijuana, Hashish, THC, Weed

## Abstract

Cannabis products (marijuana, weed, hashish) are among the most widely abused psychoactive drugs in the world, due to their euphorigenic and anxiolytic properties. Recently, hair analysis is of great interest in analytical, clinical, and forensic sciences due to its non-invasiveness, negligible risk of infection and tampering, facile storage, and a wider window of detection. Hair analysis is now widely accepted as evidence in courts around the world. Hair analysis is very feasible to complement saliva, blood tests, and urinalysis. In this review, we have focused on state of the art in hair analysis of cannabis with particular attention to hair sample preparation for cannabis analysis involving pulverization, extraction and screening techniques followed by confirmatory tests (e.g., GC–MS and LC–MS/MS). We have reviewed the literature for the past 10 years’ period with special emphasis on cannabis quantification using mass spectrometry. The pros and cons of all the published methods have also been discussed along with the prospective future of cannabis analysis.

## Highlights

- Latest trends in bioanalysis of marijuana determination in hair discussed.
- Recent approaches in hair sample preparations explained.
- Different factors affecting marijuana detection discussed.

## Introduction

For many centuries cannabis has been abused for its psychoactive properties [1]. Recently, cannabis has been rated as the most highly and widely abused illicit drug around the world [2, 3]. Cannabis is primarily banned around the world except for some countries where low doses of synthetic cannabinoids are allowed for management of pain and nausea in chronic illnesses [4]. Cannabis abstinence is a big problem around the world for cannabis users. Offenders can lose their job, driving license, incur hefty fines, and be sent to prison [5]. Abstinence from cannabis can also help to control drugs facilitated crimes, workplace drugs abuse, and toxicology [6–9]. It is crucial to tackling the false-positive scenario, where false-positive results can be due to chemically related substances (e.g., codeine in the opiate test), and interferences by some medicines (e.g., non-steroidal anti-inflammatory drugs) as shown in the literature [10]. Hair analysis complements urine and blood analyses [11, 12]. Drug detection in hair depends upon the sensitivity of the method used for analysis along with the dose of cannabis ingested. Hair detection is also dependent upon the route of drug administration, purity, duration of abuse, amount of hair sample available, pH variations due to hair dying material and metabolic rate of offenders [13, 14]. Hair analysis gives a cumulative reflection of long-term abuse. The hair allows facile storage, non-invasiveness, and no risk of infection.

This review is timely, as there is a gap in the knowledgebase, and there is no single review to discuss and compare the state of the art in hair analysis of cannabis. The novelty of this review is that it discusses all procedures and issues with sample preparation to analysis in hair matrix and it will be a single point of contact for any researcher wishing to peruse the analysis of cannabis in hair.

Hair analysis has a wider window of detection, which can range from a week to many months and up to a year or so, depending on the length of the hair strand (ideally hair grow at 1 cm per month) [15]. Segmental analysis of hair strands reveals the pattern of drug use [16]. Recently, It has also been found that in the newly formed hair, the drugs take 2 weeks to reach and deposit in hair follicles [17]. It is also suggested to collect hair from a suspected offender 1 month to 2 months after ingestion because it would have been reached and deposited in the hair securely, which would complement the results of blood, urine and saliva samples collected and analysed [18]. It has also been reported that pigmented hair binds more drugs as compared to non-pigmented hair [19, 20]. When a crime is reported late, then the conventional method of analysis is of no use except for hair analysis as it can give a retrospective drug test for past drug abuse [21].

The major metabolite of cannabis is ∆9-tetrahydrocannabinol (∆9-THC), extracted from plant *Cannabis Sativa* [22]. THC exerts its effects by interacting with cannabinoid receptors type 1(CB_1_) within the central nervous system [22]. THC has a long half-life, due to its lipophilic nature and due to its distribution to lungs, liver, spleen and adipose tissues followed by redistribution to the circulatory system for eventual metabolism. Hydroxylation of THC in the liver is catalysed, by Cytochrome P450 (CYP’s) enzyme to form psychoactive metabolites like 11-hydroxy-∆9-tetrahydrocannabinol (11-OH-THC) and further oxidation yield 11-nor-∆9-tetrahydrocannabinol-9-carboxylic acid (THC-COOH) [23]. THCOOH is usually the most sensitive peak detected in the mass spectrometer [24].

However, recent research has found that THC-COOH has a meager hair incorporation rate as well (even lower than ∆9-THC) [23] and this is why detection and quantitation of such low quantities (pg/mg or fg/mg) of THC metabolites requires specialized instruments like GC–MS/MS or LC–MS/MS etc. to detect and quantitate these low concentrations. Usually, in drug analysis laboratories, cannabis metabolites are screened using enzyme-linked-immunosorbent assays (ELISA) and confirmed using GC–MS/MS or LC–MS/MS techniques [25–28].

## Hair sample preparation techniques

For this review, online searches were conducted for THC analysis for the past 10 years. The research articles were explored online using PubMed, Google Scholar, Scopus, and ScienceDirect search engines. The parameters considered for review were hair sample collection, extraction, and sample screening and confirmation analysis.

**Fig. 1:**
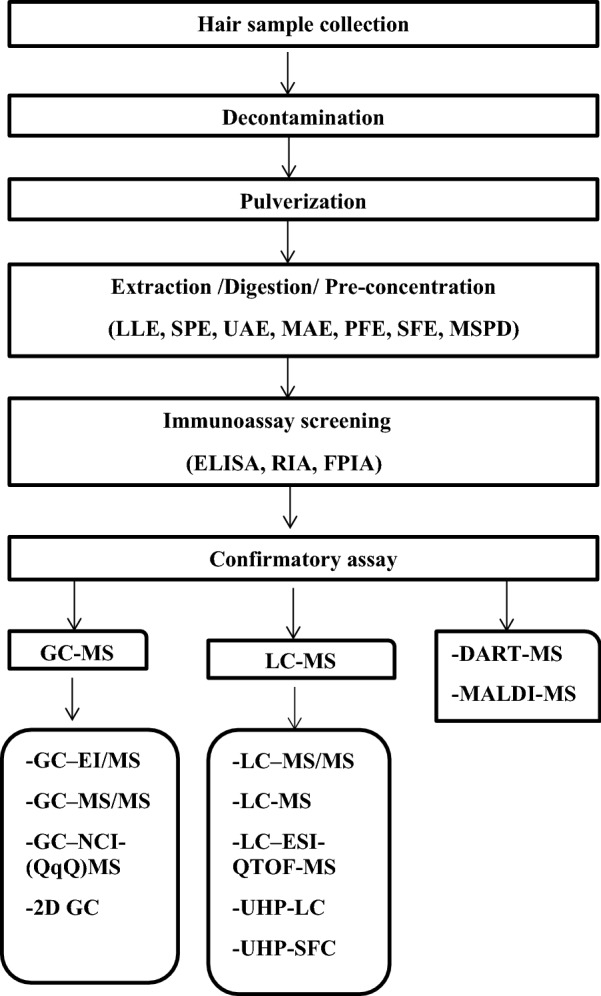
Showing the process of extraction and analysis for a THC sample

Figure 1 below showing the process of extraction and analysis for a THC sample.

### Hair sample collection

Hair samples for THC analysis should be collected 1 month after the first drug intake that is why it is one of the limitations of hair analysis, and that is why hair analysis could be used as a complementary test together with blood analysis and urinalysis [26, 29]. The correct assignment of zero value to the hair sample received in the laboratory should include the actual range of time from drug intake and collection to analysis, for segmental hair analysis [17, 30, 31]. Collection of a maximum of 60 hair fibers per sample is advised due to the risk of the oblique collection, which introduces an error with hair grown in different periods [29, 32]. Hair collection should be from cortex posterior of the head, which has fewer differences in hair size, and it has more excellent blood circulation. The hair sample should be tied up with a thread and cut as close as possible to the scalp, 20–200 mg of hair is enough for screening and confirmation of THC analysis (about the thickness of a pencil). The hair will be stored in aluminum foil, which is a secure storage tool while marking the root end, which is essential for segmental analysis [33]. Storage directly in plastic envelops/tubes can extract lipophilic substances from hair and softeners from plastic could cause contamination. So storage in sealed paper envelops in a dry and dark place is preferred along with case history and hair sample characteristics [18, 26]. For sectional analysis, the hair sample is cut into segments. One cm of hair segment from the root will give us the amount of THC utilized in last month depending on the intake of THC, considering up to 15 days for the drug to be incorporated into the hair shaft [6, 8, 30, 31, 34, 35].

### Hair sample preparation for analysis

Hair sample analysis is comprised of the following discreet steps. Hair samples are first decontaminated, followed by pulverization succeeding by digestion. This is followed by extraction methods like liquid–liquid extraction (LLE) or solid-phase extraction (SPE), and finally, the sample is concentrated and then quantified by techniques like (e.g., LC–MS/MS/GCMS, etc.).

#### Decontamination

It involves removal of environmental contaminants like (sweat, sebum, dust, cosmetics and hair colors) this is to avoid the risk of false positives (but decontamination method should not extract THC from hair matrix). For decontamination purpose, the hair sample is washed with different solvents or buffers, etc. most popular is non-protic solvent like dichloromethane (DCM) or protic solvent like methanol which do not cause the hair to swell [16, 19, 20, 36–39]. For decontamination purpose, the hair samples are soaked and washed in the above solvents a few times and then dried [40–43]. Furthermore, washing alone with isopropanol for cannabinoids can also work well [44–46]. In some methods, the decontamination of cannabinoids is performed by washing with ether followed by acetone [47] or washing with ether followed by DCM [48–50] sometimes water followed by acetone [48, 51, 52]. Some author used DCM for sample decontamination, followed by methanol and water [50, 53]. Surfactant shampoo can also be applied for decontamination of hair for cannabinoids study [54].

#### Pulverization

It is an essential next step, which comes after decontamination and before extraction. In this process, the hair sample is cut into fragments of 1–3 mm in length and milled by a dried mini-ball mill and this process helps in breaking down the hair matrix and make them into a powder which causes the release of drugs from the hair matrix very easy [44, 55].

#### Extraction/digestion/preconcentration

After sample preparation and pulverization, the next steps involve extraction/digestion and pre-concentration, as shown in Fig. 1.

These methods require the separation of analytes from the solid keratin matrix without chemically modifying them. The THC entrapped inside hair matrix are freed and released using extraction and digestion solvents like acidic or alkaline digestion solvents, extraction with a solvent, enzymatic digestion or buffer solvents incubation. Inappropriate choice of extraction method can cause degradation of THC metabolites and results in a false negative. Some modern extraction methods have also been applied to enhance the yield of THC analytes. These include microwave-assisted extraction (MAE) [56, 57], ultrasonic-assisted extraction (UAE) [58, 59], pressurized fluid extraction (PFE) [60], matrix solid-phase dispersion (MSPD) [59, 61], supercritical–fluid extraction (SFE) [62, 63] and micro pulverized extraction [64, 65].

The MAE method is most appropriate for extraction of THC because, in the MAE method, the solvent methanol will penetrate inside the hair matrix and causes the hair to swell, microwaves will break hair matrix, and this procedure will allow the lipophilic compounds to get out and dissolve in methanol.

Ultrasonic assisted extraction (UAE) uses methanol, which is the first and most common method of pre-concentration involving ultrasonic incubation for 5–18 h in 1–2 ml of methanol [66, 67]. Methanol is hydrophilic, which can penetrate hair matrix and causes it to swell and then release the analytes from hair by diffusion [26]. The mostly methanolic extract is quite clean, and it is most directly used for GC–MS analysis, but it can also cause some degree of contamination and permit low recovery of THC drugs, and further purification is necessary by LLE and SPE [68]. Methanolic extraction is very important for the analysis of cannabinoids due to poor sensitivity [54, 69, 70].

Pressurized fluid extraction (PFE) is an automated extraction procedure, which reduces the amount of solvent and time required for the extraction of THC from the matrix. PFE uses elevated temperature and pressure to increase the rate and efficiency of the extraction process.

Matrix solid-phase dispersion (MSPD) involves a simple dispersion of the THC sample constituents on the surface of appropriate solid support and subsequent elution of the THC metabolites with a suitable solvent.

Supercritical Fluid Extraction (SFE) is the process of separating the THC component from the matrix using supercritical fluids as the extracting solvent. For THC analysis, carbon dioxide is the most used supercritical fluid, modified by methanol solvent.

Micro-pulverized extraction (MPE) of hair samples are performed with adding stainless steel bullet to the vial containing hair and then centrifuging the tube at different speeds to get THC out of the hair matrix.

Liquid chromatography-time of flight mass spectrometry (LC-TOFMS) is a most advance automated screening method for cannabinoids analysis [54] a very best result was obtained by using LC-TOF–MS with methanol extraction as compare to acid extraction and alkaline digestion. The extraction of 20 mg hair specimen with methanol and then sonication in 4 ml of methanol for 8 h at 50 °C LOQ will be in the range 0.015 ng/mg [54]. A basic methanolic extraction has also been performed recently for cannabinoids extraction with LOQ of 0.1 pg/mg [70].

Acetonitrile has also been used for the extraction of THC from hair samples. Excellent results are obtained using 50 mg of hair specimen with 2 ml of acetonitrile and incubation period of 12 h at 50 °C in a thermostatic water bath and followed by LLE and then SPE analysis, which resulted in a very high THC recovery with LOQ for THC < 0.1 ng/mg and analysis performed using GC–MS. However, the extraction procedure is very cumbersome [71]. The aqueous NaOH can also be used for extraction of abused drugs from human hair. The extraction with NaOH is more advantageous for cannabinoids [49, 72–75]. Emìdio et al. also used the THC extraction with NaOH [50]. In their method, the hair matrix was digested with 1 mL of 1 M NaOH kept at 90 °C temperature for 15 min followed by headspace solid-phase microextraction (HS-SPME) and finally analysed by GC–MS [50]. Conti et al. [24] enhance the digestion and detection of Cannabis by using a combination of NaOH digestion with advance technique, i.e. surface-activated chemical ionization (SACI), electrospray ionization (ESI), mass spectrometry (SACI–ESI–MS) that got him a very high sensitivity.

Enzymatic digestion is another technique used for hair analysis. The most useful enzymes are Pronase [61, 76], β-glucuronidase, and arylsulfatase [77]. For the extraction of THC, Baptista et al. used a mixture of two-enzyme β-glucuronidase and arylsulfatase with a 2 h incubation time at 40 °C temperature, followed by LLE and gas chromatography. The advantage of this technique is the high yield of the product obtained, but the disadvantage is that it can cause the denaturation of antibodies used in the immunoassay.

For the decontamination step, several extraction solvents (either hydrophilic protic, or hydrophobic) and reagents are used, but there is no universal procedure present that will give optimum results. For the extraction of THC from hair, methanol is widely used in the literature, but methanol may also cause contamination, which could affect the sensitivity of LC–MS assay. That is why the filtration step is very important before the sample injecting into the LC–MS system. Apart from methanol, acetonitrile, aqueous solution of NaOH and certain enzyme could also require filtration to make it more cleanly for LCMS analysis.

It is known that cannabis concentration in hair is usually very low, and hair matrix contains several contaminants, so a further pre-concentration step is required to enhance the yield of our desire drug and to remove contaminants. That is why after extraction and before doing a major assay of chromatography, we must do pre-concentration step using SPE or LLE, etc. for THC analysis. For cannabis study, LLE procedure is highly preferred [33, 53, 71]. Recently, a very advanced technique has also been proposed for sample pre-concentration like headspace solid-phase dynamic extraction (HS–SPDE) technique, headspace solid-phase microextraction (HS–SPME) techniques and Supercritical Fluid Extraction (SFE). Among them, HS–SPME, can give complete retrieval of drugs more than 50% compared to SPME, while SFE is good but somewhat expensive.

Pre-concentration is next step for sample extraction and cleaning, which further enhance the detection and yield of cannabinoids. The most common method used for sample pre-concentration is liquid–liquid extraction (LLE) and solid phase extraction (SPE). For the drugs of abuse, several new advanced techniques have also been developed like molecularly imprinted polymers (MIP) that can eliminate a matrix interferences [78, 79]. More recently, new sophisticated methods which use organic solvent in low volume for sample pre-concentration like fully automated headspace solid-phase dynamic extraction (HS-SPDE) [74], micro-extraction by packed sorbent (MEPS) [80], headspace liquid-phase microextraction (HS-LPME) [52] and hollow fiber liquid phase micro-extraction (HF-LPME) [52]. However, overall for cannabinoids, an LLE procedure is preferred for sample cleanup [47].

## Hair analysis techniques

There are at least 113 cannabinoids identified in cannabis. Few of the Cannabis metabolite are as follows. Tetrahydrocannabinol (THC), Cannabidiol (CBD), Cannabinol (CBN), and 11-nor-9-Carboxy-THC (THC-COOH) and 11-Hydroxy-Δ9-tetrahydrocannabinol (11-OH-THC), etc. However, among the THC is the most common psychoactive component [47, 81]. These metabolites are in the very low concentration found in hair due to the acidic nature of hair and weak bonding to melanin pigments. They are present in the range of Femto to Picogram per milligram of human hair. So it is great challenge to detect cannabinoids in hair. A number of analytical procedures that have been developed and employed for the quantitation of THC metabolites, starting from the end of the year 1990. These include immunoassays (IA), gas chromatography-mass spectrometry (GC–MS), liquid chromatography-mass spectrometry (LC–MS) [18]. We have investigated the most recent and relevant literature regarding cannabis determination in hair. In the given Table 1, we have summarized all the analytical approaches used for cannabis quantitation, and in the following section, we discuss the detail of all these analytical procedures. There are three major assays used for cannabis analysis in hair.

**Table 1:** A comparative analysis of methods for Cannabis determination

Drugs	Amount of hair	Decontamination	Extraction protocol	Instrument	Linear range	References
THC	50	DCM	LLE (MeOH)	UHPLC–MS/MS	6–27	[53]
THC	50	DCM	LLE (*n*-hexane/ethyl acetate) (90:10, v/v)	UHPLC–MS/MS	0.02–5.4	[33]
THC	50	DCM	Ball mill	LC–MS/MS	30	[28]
THC	50	DCM	Digestion(ACN)	LC–MS/MS	0.2–50	[71]
			LLE (*n*-hexane/ethyl acetate, SPE (Strata-X)			
THC, THC-COOH	20	MeOH	Ethyl acetate	LC–MS/MS	1	[27]
THC	20	Nil	ACN/MeOH/20 mM ammonium formate buffer pH 3(10:10:80, v:v:v)	LC–MS/MS	2–82	[82]
THC-COOH, THC	50	Water, acetone	MeOH	LC–MS/MS	2.5–20	[83]
THC-COOH, THC	50 20	Petroleum ether	MeOH MeOH	LC-MS/MS LC-TOF	2.5–20 5–75	[83] [54]
THC-COOH, THC Cannabinoids	50 20	Shampoo, water, acetone	MeOH MeOH	LC-MS/MS LC-TOF	2.5–20 5–75	[83] [54]
THC	20	Water, acetone	MeOH/ACN/water/2 mM ammonium formate (25:25:50, v/v/v)	LC-TOF	3–15	[84]
THC	50	DCM	Enzymatic digestion, LLE (pentane)	GC–MS	15–20	[85]
THC	10	Water, acetone	Digestion (1 M NaOH), Derivitisation MSTFA	GC–MS	10–120	[52]
THC	10–20	Water, acetone	Digestion with 1 M NaOH Derivatization with BSTFA + 1% TMCS	GC–MS	15–30	[48]
THC	10	Petroleum ether	Digestion with 1 M NaOH	GC–MS	70	[86]
THC-THCA-A	30–50	Water, acetone, petroleum ether	Digestion with 1 N NaOH LLE with *n*-hexane/ethyl acetate (9:1, v/v) derivatization with MSTFA	GC–MS	20–50	[47]
THC	10	Petroleum ether, water, ACN	Digestion with 1 M NaOH	GC–MS	7–31	[50]
THC-COOH	25	Isopropyl alcohol	LLE with *n*-hexane/ethyl acetate (9:1, v/v) Derivatization with PFPA/PFPOH	GC-NCI-MS	0.02	[46]
THC-COOH, THC	20–50	Nil	Digestion with 1 M NaOH LLE with *n*-hexane/ethyl acetate (9:1, v/v)	GC-NCI-MS	0.01	[87]
THC-COOH	20	DCM	SPE (Bond Elut Certify) Derivatization with TFAA + HFIP	2D GC–NCI–MS	0.05	[49]
THC	Single hair	DCM	Nil	Nil	Nil	[88]
THC	20	Shampoo, deionized water, acetone	Methanol methanol/water(1:1, v:v)	LC–TOF	0.0125	[54]
THC	50	Water, petrol ether, methanol	Methanol, SPE, derivatization MTBSTFA	GC–MS	0.1-LLOQ	[89]
THC	50	Dichloromethane	Methanol, hexane/ethyl acetate (90:10, v:v) derivatization HFBA/ethyl acetate	GC–MS	Nil	[69]
THC		Acetone, petroleum ether	Methanol, HS SPE	HPLC–ED	300	[89]
THC-COOH	15	Nil	Methanol, SPE Narc-1 SP cartridge Pentafluoropropanol/PFPA	GC–MS/MS	0.1 LLOQ	[70]
THC	50	Dichloromethane	Acetonitrile, LLE (hexane:ethyl acetate (55:45, v:v)) SPE: Strata-X cartridge	LC–MS/MS	0.05	[71]
THC	10	Nil	Enzyme-linked immunosorbent assays	Immunoassay test	100	[90]
THC-COOH	30	Water, acetone, methanol, dichloromethane, phosphate buffer	0.1 M NaOH, SPE (Waters Milford)	LC/SACI-ESIMS/MS–MS	Nil	[24]
THC-COOH	20	Methanol	NaOH, LLE (*n*-hexane:ethyl acetate 9:1, v:v) PFPA/PFPOH	GC-NCI-MS	0.025 ng/mg	[91]
THC,THC-COOH	20	Methanol	NaOH, LLE (*n*-hexane:ethyl acetate 9:1, v:v) PFPA/PFPOH	GC-NCI-MS	THC: 2.5 ng/mg THCCOOH: 0.025 ng/mg	[92]
THC-COOH		Methanol	NaOH, LLE (*n*-hexane:ethyl acetate 9:1, v:v) PFPA/PFPOH	GC-NCI-MS		[44]
THC, THC-COOH	30–50	Water, petrol, ether, acetone	NaOH, LLE (*n*-hexane:ethyl acetate 9:1, v:v) MSTFA	GC–MS	0.02 ng/mg	[47]
THC	10	Petroleum ether, deionized water, dichloromethane	NaOH, SPME (PDMS fiber)	GC–MS/MS	0.031 ng/mg	[93]
THC	10	Petroleum ether, deionized water, dichloromethane	NaOH, HF–LPME	GC–MS/MS	0.015 ng/mg	[50]
THC	10	Water, acetone	NaOH, NaCl, SFME, deriv. MSTFA	GC–MS	0.012 ng/mg	[52]
THC-COOH	25	Isopropyl alcohol	NaOH, LLE (*n*-hexane:ethyl acetate, 9:1, v:v) PFPOH/PFPA	GC–MS/MS	0.02 ng/mg	[46]
THC	15	Deionized water, acetone	NaOH, SPME	GC–MS	0.012 ng/mg	[48]
THC	50	Dichloromethane	NaOH, LLE (*n*-hexane)	GC/MS	0.025 ng/mg	[42]
THC	50	Isopropyl alcohol	NaOH, LLE (*n*-hexane–ethyl acetate, 75:25 v:v)	GC–MS	0.006 ng/mg	[44]
THC-COOH	25	Isopropyl alcohol	NaOH, LLE (*n*-hexane–ethyl acetate 9:1 v:v) PFPOH/PFPA	GC–MS/MS	0.015 ng/mg	[45]
THC	10	Deionised water, petroleum ether, dichloromethane	NaOH, SPME, MSTFA	GC–MS	0.05 ng/mg	[94]
THC		Deionised water, petroleum ether, dichloromethane	NaOH, HS-SPDE, MSTFA	GC–MS	0.14 ng/mg	[95]
THC		Dichlorometane	NaOH	GC–MS	0.1 ng/mg	[73]
THC-COOH	20	Dichlorometane	NaOH, SPE(*n*-hexane–ethyl acetate, 75:25 v:v)	GC–MS	0.3	[72]
THC-COOH		Nil	NaOH, hexane/ethyl acetate	GC–MS	0.3	[96]
THC, THC-COOH		KH_2_PO_4_ 1 M, water, methanol	Hexane/ethyl acetate, TMSI/HFIP/PFPA	GC–MS/MS	0.1–1	[97]
THC-COOH	50	Nil	NaOH, LLE (*n*-hexane–ethyl acetate 9:1 v:v) BSTFA	GC–MS/MS	Nil	[98]
THC-COOH	20	Dichloromethane	NaOH, SPE (*n*-hexane–ethyl acetate, 75:25 v:v) TFAA/HFIP	GC–MS/MS	0.05 LLOQ	[49]
THC-COOH	20	Methanol	NaOH, LLE (*n*-hexane–ethyl acetate 9:1 v:v) PFPOH/PFPA	GC–MS	0.025 ng/mg	[92]
THC-COOH	20	Dichloromethane	NaOH, SPE (*n*-hexane–ethyl acetate, 75:25 v:v) TFAA/HFIP	GC–MS	Nil	[72]
THC-COOH	50	Dichloromethane	NaOH, LLE (n-heptane-ethyl acetate 9:1 v:v) PFPOH/PFPA	GC–MS/MS	0.05 ng/mg	[99]
THC	50	DCM	MeOH	UHPLC–ESI–MS/MS (QqQ)	6–27	[53]
THC	50	DCM	LLE with *n*-hexane and ethyl acetate SPE	LC–ESI–MS/MS (QqQ)	0.2–50	[71]
THC, THC-COOH	20	Washing sonication MeOH	LLE ethyl acetate	LC–ESI–MS/MS (QqQ)	1	[42]
THC, THC-COOH	50	Water acetone petroleum ether	MeoH	LC–ESI–MS/MS (QTrap)	LOQ 2.5–20	[83]
THC	50	DCM	LLE pentane	GC-EI/MS	15–20	[85]
THC	15–30	Water acetone	HS-SPME	GC-EI/MS	10–20	[52]
THC	10	Petroleum ether	HS-SPME	GC-EI/MS	70	[86]
THC	10	DCM water petroleum ether	HS-SPME	GC-EI/MS/MS (IT)	7–31	[50]
THC-COOH	25	Isopropyl alcohol	LLE *n*-hexane/ethyl acetate	GC–NCI–MS/MS (QqQ)	0.02	[46]
THC THC-COOH	20-50	NaoH	LLE *n*-hexane/ethyl acetate	GC–NCI–MS/MS (QqQ)	0.01	[87]
Δ-9-tetrahydrocannabinol		DMC	LLE *n*-hexane/ethyl acetate	DART-MS LC/MS/MS	Nil	[88]
THCA-A, THC		Water acetone, petroleum ether	methanol	LC–MS/MS MRM mode	2.5 pg/mg 20 pg/mg for THC	[51]
THCA-A, THC		Water acetone , petroleum ether	alkaline hydrolysis, followed by LLE	GC–MS monitoring (SIM) mode analysis	LOD (THCA A) 0.05 ng/mg LOD (THC) 0.02 ng/mg	[47]
THCA-A and THC		Water acetone, petroleum ether	2 mL MeOH	(LC–MS/MS)	n 1–2.5 pg/mg THCA-A 5–10 pg/mg for THC	[51]
THC-COOH THC THCA-A	1–2 mm	Water acetone	MeOH	LC–MS/MS GC–MS HS–SPME–GC–MS	1.6–36 0.71–3.27 0–2.75	[51]
THCCOOH THC THC-OH	10 mg	Chloroform/isopropanol	MeOH solid phase extraction	GC–MS/MS	0.05 ng/mL, 0.004 ng/mL	[100]
THC-COOHTHC and THC	200-mg	Methanol in an ultrasonic bath	LLE ethyl acetate NaOH vertex	LC–MS/MS s	0.1 ng/mL	[27]
THC-COOH	25 mg	Methyl alcohol	Amiodarone VMA-T M3 reagent	UHPLC–MS/MS	0.09 pg/mg	[101]
Δ-9-THC, cannabidiol cannabinol	25 mg	Methyl alcohol Diethyl ether	VMA-T M3 reagent	UHPLC–MS/MS	0.01 to 0.1 ng	[101]
THC	60 mg	Methanol SDS wash	LLE *n*-hexane/ethyl acetate	UPLC SEM	0.02	[45]
THC CBN CBD	0.5 g	Mechanical stirring DMC Ultrapure water	LLE HPLC	HPLC LC/MS/MS	Nil	[102]
THCCOOH	20 mg)	Methanol	1 M NaOH acetic acid *n*-hexane:ethyl acetate LLE	LC/MS/MS	LOQ (2 pg/20 mg hair)	[103]
THC, CBN, CBD		2.0 M sodium hydroxide	Liquid–liquid extraction with hexane/ethyl acetate	HPLC–MS/MS	0.25, 0.21, and 0.22 ng g	[76]
Synthetic cannabinoids	100 mg	0.5 M sodium hydroxide	Liquid–liquid extraction with hexane/ethyl acetate	1260 HPLC-Q-TOF	10	[104]
THCCOOH THC	20 mg	1 M sodium hydroxide	Liquid–liquid extraction with hexane/ethyl acetate	GC/MS/MS-NCI	Nil	[105]
THC, CBD, CBN	50–100 mg	Water, petrol ether, methanol	SPE	GC–MS	0.1 pg/mg	[70]
THC, CBD, CBN	20 mg	Shampoo, deionized water, acetone, air-dried overnight	1 mL methanol/water (1:1 v1:v1) filtered 0.45 PTFE syringe filter	LC–TOFMS	Nil	[54]
THC, CBD, CBN	50 mg	Dichloromethane	LLE	GC–MS	Nil	[69]
THC, CBD, CBN		Acetone, petrol ether	Ultra sonication	HPLC–ED	300 (ng/mg)	[89]
THC and THC-COOH	20 mg	Washed in methanol for 30 s	Acidified methanol (2 mL) for 18 h at 45 °C	LC–MS/MS	LoQ of 75 pg/mg	[106]
11-Nor-9-carboxy-THC		Methanol/water wash follow NaOH digest at 75 °C	Hexane/ethyl acetate liquid extraction	HPLC SCIEX 6500 + QTRAP system	0.2 pg/mg	[107]
THC], THC-OH, THC-COOH	segmented in 1 cm	Methanol chloroform/isopropanol	Ultra sonication SPE	GC–MS/MS	0.05 ng/mL	[108]
THC, CBD, CBN		Sodium hydroxide solution	Liquid–liquid extraction (LLE)	GC–MS/MS	0.01 ng/mg	[109]
THC	20 mg	methanol	Acidified methanol 18 h at 45 C	LC–MS/MS.	75 pg/mg	[106]
THC, CBN		Water, acetone, NaOH	Solid-phase extraction	GC–MS/MS		[110]
THC-COOH	10 mg	0.1% sodium dodecyl sulfate, water, and methanol	Micro pulverized extraction (MPE)	(LC/MS/MS	0.2 pg/mg	[55]
Cannabis	30 mg	Tween 80, Acetone	Methanolic incubation Sonication	LC-HRMS	2 to 30 pg/mg	[111]
Cannabis		Methanol	6-h ultrasonic-assisted methanolic extraction	GC–MS/MS		[112]
Synthetic cannabinoids		Samples were fortified by soaking	Pressurized liquid extraction (PLE) SPE	HPLC-HRMS	9 to 40 pg/mg	[113]
THC CBN CBD	3 − 6 cm 100 mg	*n*-hexane Acetone	LLE *n*-hexane/ethyl acetate	GC–MS SIM	0.01 0.06 0.03	[109]
D9-THC	50 mg	0.1 N HCl NaOH 2 M	Liquid–liquid extraction	GC–MS ELISA		[114]
THC, CBN, CBD	5 cm	Water methanol	MALDI-MS/MS	Quadrupole LC–MS/MS		[115]

i. Immunoassays (IA).
ii. Gas chromatography–mass spectrometry (GC–MS).
iii. Liquid chromatography–mass spectrometry (LC–MS).

### Immunoassays (IA)

Immunoassays are the most simple and easy tests for screening of drugs in the hair matrix. Following are the major types of immunoassay techniques.

- Enzyme-linked immunosorbent assay (ELISA) technique.
- Fluorescence polarization immunoassay (FPIA) technique.
- Radioimmunoassay (RIA) technique.

These Immunochemical screening assays are very important because they rapidly analyse the specimen and screens them for drugs without the need for using highly expensive mass spectrometric techniques. Among these immunoassays, the Enzyme-linked immunosorbent assays (ELISA) are considered, the most sensitive heterogeneous assays. In general, these assays are used as a preliminary assay for THC screening as it is an easy way to determine the positive samples quickly. However, while doing a direct GC–MS or LC–MS method could result in a false negative or false positive result due to certain reasons like structurally related drugs, isobar or any artifact, i.e. detergents or any other surfactant that may affect the pH of the drug sample and it will cause a variation in result. Once the screening test identifies the positive samples, then it is of great importance to confirm these positive result with a ‘gold standard’ assays like GC/MS or LC/MS [18].

Immunoassay techniques is a semi-quantitative essay which has good proficiency in producing good results, and it could also be used for determination of cannabinoids in hair matrix. The THC which had been oxidized to other cannabinoids over many months could also be recognized by ELISA assay [90, 114].

Universally, an ELISA screening procedure is combined with a GC–MS technique a confirmation technique for detection of cannabinoids in human matrix samples. This method was also adapted and validated for cannabinoids in human hair [116].

### Gas chromatography-mass spectrometry (GC–MS)

GC–MS is a standard Gold technique for the analysis of drug of abuse, particularly for THC and its metabolites in hair samples as seen in Table 1. However, GC–MS technique has some drawbacks as well. In GC–MS technique derivatization of the drug, metabolites are mostly required before injecting the sample into a GC–MS column. This additional derivatization step is time-consuming laborious, and it complicates the whole process. Derivatization refers to chemical modification to generate derivatives of the parent drugs. Joanna Znaleziona et al. [117] proposed her view on derivatisation that many of synthetic cannabis and its primary product are mainly organic molecules which have multiple active polar functional groups that are necessary for derivatization steps. Moreover, these steps improve the stability of cannabinoids and further enhance the efficacy and detectability of these compounds [117]. On the other hand, many researchers consider the LC–MS technique as a preferred choice because no such additional derivatisation steps are needed for analysis. Some other interesting studies and researchers have found that GC–MS is a cheaper and sensitive then LC–MS for the drug of abuse [18]. In Table 1, we have summarized the literature regarding all the important GC–MS methods published.

There are number of GC–MS methods developed that use different types of ion sources as given in Table 1 [77], like gas chromatography/mass spectrometry operating in electron impact mode (GC/MS-EI), gas chromatography/mass spectrometry in negative chemical ionization mode (GC/MS-NCI), gas chromatography/tandem mass spectrometry in negative chemical ionization mode (GC/MS/MS-NCI), gas-chromatography/tandem mass spectrometry in positive chemical ionization mode (GC/MS/MS-PCI), gas chromatography with negative chemical ionization using triple quadrupole mass spectrometry mode GC–NCI-(QqQ)MS/MS, and two-dimensional gas chromatography (2D GC). All these methods have their advantages and disadvantages among them electron impact (EI) ionization is more commonly used because it enhances specificity, sensitivity, and improved the limit of detection (LOD). Merola et al. [52] used a GC–EI/MS method with SPME for a number of drugs of abuse in human hair, including THC. Recently, SPME gained more interest in forensic analysis as compared to conventional SPE methods. In SPME a N-Methyl-N-(trimethylsilyl)-trifluoroacetamide (MSTFA), N,O-Bis(trimethylsilyl)trifluoroacetamide (BSTFA) and poly dimethyl siloxane (PDMS)-coated fiber are used as derivatizing agent along with electron impact (EI) ionization mode for good sensitivity. The LOD and LOQ achieved for THC were 10 and 20 pg/mg, respectively for THC.

Although the above method is very sensitive but may be affected by certain factors like sample handling procedure, sample contamination, sample carryover, and lifetime of hair fiber and time of drug consumption [52].

Kim et al. [46] developed a GC–NCI-(QqQ)MS/MS method for the detection of THC-COOH in human hair matrix, and he found that the concentration of THC-COOH is even lower than the parent THC drugs. This method uses alkaline digestion for a 25 mg hair sample, and it employs an LLE method for sample pre-concentration along with a more advanced technique HS-SPME. They used pentafluoropropionic anhydride/pentafluoropropanol (PFPA/PFPOH) and pentafluoropropionic anhydride/Pentafluoropropionic for derivatization steps. Then the proposed method used Negative chemical ionization (NCI) with QqQ MS/MS detection, which offers additional sensitivity because of the secondary fragmentations [50]. The LOD and LOQ obtained were 0.02 and 0.05 pg/mg, respectively. Because of high sensitivity, this assay was considered as more suitable for the quantitation and identification of THC-COOH in human hair.

Moore et al. [9] described a new advance technique two-dimensional gas chromatography (2D GC) coupled to MS with NCI mode for the determination of THC-COOH which has more advantages over the conventional one-dimensional gas chromatography (1D GC) over the two-dimensional gas chromatography (2D GC) which improve the peak shape. This enhances the sensitivity level and improves the peak shape in the chromatogram for structural analysis. Some studied have suggested that 1D GC is not good enough as it cannot resolve the structurally similar cannabis compounds [22]. Hence, hyphenated analytical instruments like 2D GC, LC–MS, GC–MS are more perfect in the evolution of best sensitive methods for cannabis analysis [22]. Moore et al. [49] used alkaline digestion for cleaning the hair samples followed by pre-concentration of the hair specimen by SPE method. 1,1,1,3,3,3-hexafluoro-2-propanol (HFIP) and Trifluoroacetic anhydride (TFAA) were used for derivatization of cannabinoids. The LOD was 0.05 pg/mg, which was very sensitive, and it helped to reduce the contamination issues. Two serial GC columns helped in reducing the matrix background effects and improved specificity. Deuterated analogues were used as an internal standard in this study for a standardised assay [18].

**Fig. 2:**
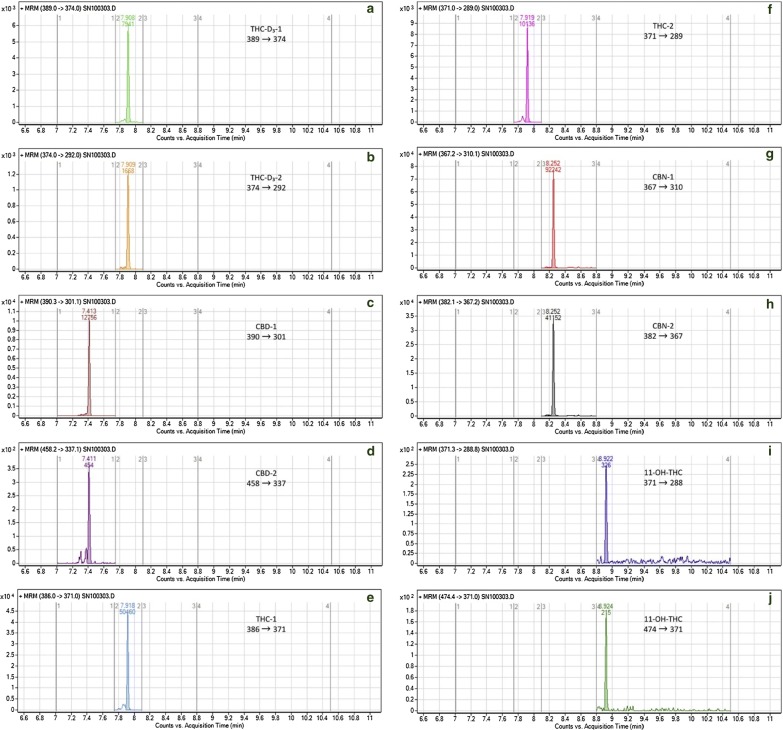
MRM chromatograms relative to a hair positive sample from a cannabis user, showing the MRM transitions applied. **a**, **b** THC-D_3_, **c**, **d** CBD, **e**, **f** THC, **g**, **h** CBN, **i**–**l** 11-OH-THC (reproduced with permission from Elsevier, Ref. [118])

Emídio et al. [50] used GC–MS/MS combined with HS-SPME for the determination of THC metabolites like CBD and CBN in human hair. A 10 mg of hair specimen was used along with a combination of headspace solid-phase microextraction (HS-SPME) with gas chromatography linked with tandem mode mass spectrometry (GC–MS/MS). HS-SPME and together with the use of tandem MS/MS offers excellent selectivity and sensitivity for cannabinoids. Because The LOQ found in this study was 0.062 ng/mg of hair, for THC and it was even below the cut-off value (LOQ ≤ 0.1 ng/mg), but this method has some limitation that it is inappropriate for the detection and quantification of THC-COOH level [18]. Eunyoung et al. [92] presented a GC/MS/MS-NCI method, which gives an enhanced sensitivity for THC-COOH in hair. This analytical procedure can be used for simultaneous detection of both metabolites of cannabis, i.e. THC-COOH and THC in human hair. The sample was extracted by LLE derivatised by pentafluoropropanol (PFPOH) and pentafluoropropionic anhydride (PFPA) and finally analyzed by GC/MS/MS-NCI assay. The concentrations of THC-COOH and THC in hair ranged from 60.41 to 7.52 ng/mg and from 0.10 to 11.68 pg/mg, respectively. LLE and GC/MS/MS-NCI combination are believed and tested to be the first best technique, which yields an excellent sensitivity level for THC-COOH. A representative chromatogram of GC–MS is shown in Fig. 2.

### Liquid chromatography-mass spectrometry (LC–MS)

Liquid chromatography (LC) is a gold standard for the separation and detection of a wide variety of drugs of abuse in hair matrices while the use of chromatography creates a three-dimensional spectrum that is valuable for the determination of structure, molecular weight, quantity, purity, and identity of drugs in hair specimens. Most of the abuse drugs like THC are polar molecules that can be easily analyzed by LC as compared to GC, and no derivatisation is required. So LC is considered a fundamental technique for toxicological analysis of drug of abuse [18]. Liquid chromatography-mass spectrometry (LC–MS) has high applicability, selectivity, and sensitivity as compared to GC–MS methods because LC–MS does not require a time-consuming step of derivatisation.

Cannabinoids and its metabolite have very low incorporation in hair, and usually, these are found in very low concentrations in hair, i.e. pg/mg. Mercolini et al. [27] have also developed an innovative LC–ESI-(QqQ)MS/MS method for determination of THC and THC-COOH in human hair matrix. This method is very sensitive because it has a LOD of 1 pg/mg of hair for all metabolites of cannabis. This analytical technique also has some limitations because the concentration of THC-COOH is very low as compared to the parent drugs, so this may cause an error and give false-positive results for cannabis [27].

Lendoiro et al. [71] used LC–ESI-(QqQ)MS/MS for the screening of 35 multiclass of drugs include THC. This multi-analytes screening method reduces the cost, the amount of specimen used, and save time. For all the multi-drug analysed the LOD were 0.2–50 pg/mg, which is less than that reported in the Society of Hair Testing (SoHT) guidelines [71]. All the positive result were then confirmed by a second injection of the same specimen. This assay is also very suitable for routine toxicological analysis.

Mercolini et al. [27] used LC–MS/MS, with a reversed-phase column for the determination of THC-COOH and THC in chronic drug abusers. The developed acquisition method for mass spectrometry was multiple reaction monitoring (MRM) mode, and electrospray ionization (ESI) mode was used for ions source. GC method here is time-consuming and expensive. The limits of quantitation and detection were 3 pg/mg and 1 pg/mg, respectively, for both analytes.

The more advanced and rapid technique was introduced by Di Corcia et al. [53] by separating thirteen drugs of abuse including THC within a very small time window of 5.5 min plus 2.5 min of column re-equilibration time. First, the THC is extracted with an organic solvent and then directly injected into a UHPLC–ESI-(QqQ)MS/MS system. The ultra-high pressure liquid chromatography (UHPLC) is a very specific technique because it produces a very narrow peak that may reduce the likelihood of unwanted interferences. This method is highly specific and accurate because the LOQ (20 to 80 pg/mg) achieved were lower the cut-off values that are given by the society of hair testing (SoHT) [71]. The UHPLC is a very rapid technique making them suitable for routine toxicological analysis [33, 119]. Pichini, Simona, et al. used UHPLC–MS/MS for the detection of new cannabis metabolites, i.e. THC-COOH-glucuronide in 25 mg of the human hair matrix. For the extraction of THC-COOH-glu, involve buffered digestion followed by chromatographic mass spectrometry. The LOQ obtained for THC-COOH-glu was 0.25 pg/mg. To the best of our knowledge, UHPLC–MS/MS was the first chromatographic techniques used for the quantification and detection of THC-COOH-glu in human hair specimens. Also, THC-COOH-glu is a new biomarker which is used in confirming the specificity and sensitivity of this new hair biomarker in cannabis smokers [101].

Salomone et al. [33] also described a UHPLC–ESI-(QqQ)MS/MS protocol for detection of synthetic cannabinoids groups. The method was fast, simple, and sensitive because the chromatographic run time was 9 min, and all the synthetic cannabinoids were eluting between 2.2 and 5.5 min. The LOQ of this assay ranged between 0.7 pg/mg to 4.3 pg/mg, respectively.

Unfortunately, there is also certain limitation of this technique that the number and type of synthetic cannabinoids are growing every month and new cannabinoids are coming to the market every day. This is the reason that the identification of individual drugs become difficult and also pure standards and fragmentation spectra are also hardly available [33]. Dulaurent et al. [120] first time introduce Quadrupole/Quadrupole/Ion Trap(QTRAP) mass spectrometer (a hybrid API 5500 AB Sciex, Courtaboeuf, France) system for the quantification and detection of cannabis. The methods were highly specific and sensitive, and the LOD for cannabis was 0.2 pg/mg. The application of LC–MS/MS system like AB SCIEX API 5500 QTRAP LC–MS system has a broader role and more applications then GC–MS. The intra-assay precision and accuracy were assessed using quality control samples at 0.2, 0.5, 2.0 and 20 pg/mg for THC-COOH and quality controls at concentration 50, 100, 500 and 10,000 pg/mg for THC, CBD, and CBN [120]. At the same year [28] Koster et al. used triple quadrupole mass spectrometry LC–(QqQ)-MS/MS, with ball mill sample grinding technique for fast sample preparation and detection of cannabis in hair specimen. In LC–(QqQ) MS/MS, analytical techniques, they detect all 17 abused drugs in a single injection of the specimen within a run time of 4.8 min [28].

Míguez-Framil et al. [102] proposed a high-performance liquid chromatography with tandem mass spectrometry (HPLC–MS/MS) method for the fast detection of cannabis metabolites in hair matrix such as CBN, Δ9-THC, and CBD. Míguez-Framil et al. [102] design a new stationary phase for the column which has smaller length and small particle size than previous columns. All this reduced the time of chromatographic separation to less than 15 min. The methods were very faster as compared to conventional methods like GC–MS/MS, GC–MS, and LC–MS/MS procedures. The limits of detections were 0.25, 0.22, and 0.21 ng/g, for Δ9-THC, CBD, and CBN, respectively. The relative standard deviation of inter-day and intra-day precision was lesser than 7% and 4%, respectively, and the analytical recoveries were between 95 and 106%, and 93 to 105%, respectively, according to the FDA guidelines, there is a ± 15% error window [102]. Liquid Chromatography-Electrospray Ionization-Quadrupole Time Of Flight-Mass Spectrometry (LC–ESI-QTOF-MS) is a new advanced technique for the detection and quantitation of drugs of abuse like many synthetic cannabinoids, phenethylamines, and cathinone. This method is highly applicable for drugs of abuse analysis of human hair in toxicological studies [104]. This method was introduced by [104] Gottardo et al., which quantitates based on the accurate masses and isotopic detection of compounds with the help of QTOF analyzer. QTOF analyzer carries out full-scan acquisitions with high mass accuracy and superior sensitivity that could be integrated with fragmentation data, which is finally recorded in a very high resolution. These features, in QTOF, makes it possible to find out several chemical compounds without monitoring their pre-defined masses. All these qualities make QTOF mass analyzer a valuable tool for toxicological screening of specimen samples. Although QTOF-MS technique is an ideal tool for quantitative analysis of compounds but there is some limitation, due to a the constant increase in number of new psychoactive compounds coming to the markets worldwide and these new compounds and their fragmentation spectra and standards are scarcely existing thus limiting the application of this assay [104].

**Fig. 3:**
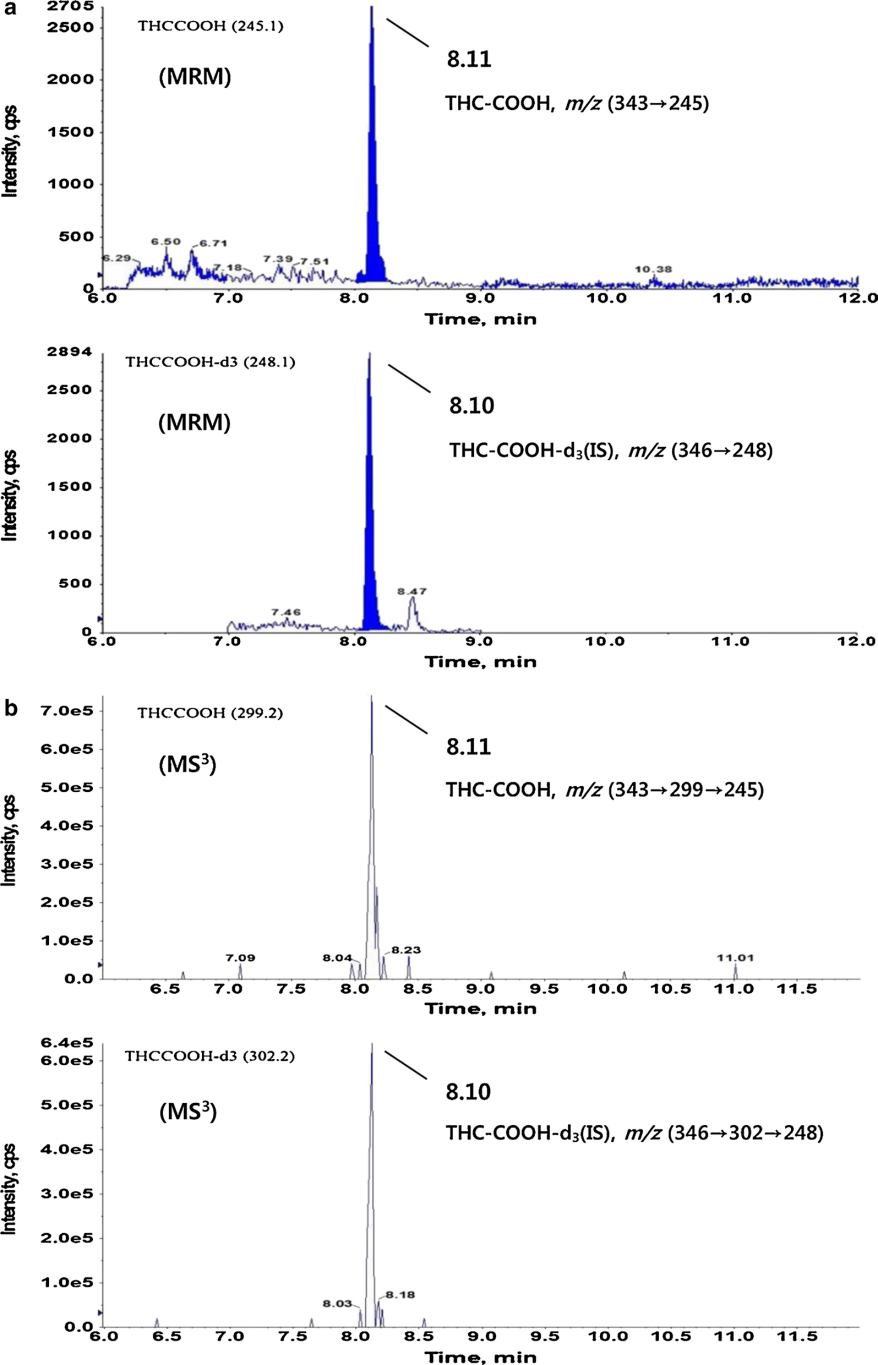
Representative chromatograms of THC-COOH and THC-COOH-d3 in an authentic hair sample of MRM (**a**), and MS3 (**b**) modes (reproduced with permission from Elsevier, Ref. [122])

New and more sophisticated methods continued to emerge, the one method invented by [121] Breitenbach et al. This method comprised of an ultra-high-performance supercritical fluid chromatography (UHP-SFC) technique which is used for the determination of synthetic cannabinoids. UHP-SFC and ultra-high-performance liquid chromatography (UHP-LC), both could be used to analyzed non-volatile, thermally labile and polar compounds without the need for sample derivatization as usually required in gas chromatography method. However, UHP-SFC has mobile phases that are more diffusive with low viscosity as compared to UHP-LC. UHP-LC may enhance the separation speed up to four-time faster as compared to UHP-LC with similar resolving capacity. Furthermore, UHP-SFC can also work more efficiently in a normal phase mode so that it could produce exceptional selectivity for many structurally related biomolecules [121]. Breitenbach et al. also compare UHP-SFC with GC and UHP-LC for determination of synthetic cannabinoids. They have got a very accurate and sensitive result using UHP-SFC as compare to GC and UHP-LC. UHP-SFC resolved 11 compounds in just a 10 min gradient in a compound mixture while for a similar compound mixture, GC determined 18 compounds in a 24 min gradient run and finally for UHPLC the gradient run time was 13 min both GC and UHP-LC had the same resolution ≥ 1 [121]. Three quadrupoles are used in MS3 scans (Quad 1, Quad 2, and Quad 3) and these three are lined up in a row. The LCMS/MS system used in the following figure is a triple quadrupole mass spectrometer where the Precursor ions are selected in Quad 1 and transferred to Quad 2 for fragmentation this is followed by sending the fragmented mass to Quad 3 for mass scanning (Fig. 3).

## Novel approaches in Hair THC analysis

In current years, many advanced and sensitive analytical methods have been introduced that gave highly specific and accurate results for drugs in human hair. Example include the matrix-assisted laser desorption/ionization mass spectrometry (MALDI–MS) technique and Direct Analysis in Real Time mass spectrometry (DART-MS) technique. A MALDI-MS imaging technique is a highly innovative procedure as compared to conventional LC/MS and GC/MS analysis for cannabinoids metabolites determination. This method is highly superior as compared to old conventional methods because of its proficiency in a smaller window of detection. Small volumes of samples and easy and robust preparation methods are needed to give a full chronological data of drugs in a single hair. MALDI-MS has been used with high efficiency for THC and related drugs in hair [115]. However, it has few limitations like it cannot differentiate between the isobaric CBD and THC and it has shown reduced ionization efficacy of the drug, but the only derivatisation with N methyl-pyridium can enhance ionization that could improve the sensitivity and detection of THC metabolites. (MALDI-MS) profiling and imaging have been successfully used for the detection of cannabinoids in a single hair sample, and additional research is required in this domain [88]. DART-MS is the second most rapid method for detection of drug of abuse in hair that does not require a time-consuming sample preparation step. DART-MS is a new approach for determination of THC in human hair that is based on the use of helium heated gas that desorbs a desired ions of drugs from surface of the specimen at very high temperature [88]. DART-MS has demerits that it cannot distinguish between isobaric species like CBD and THC, and it requires a large amount of specimen sample, and the LOD is around 5 ng/mg. Although DART-MS has low sensitivity, but it can be used with great efficacy for screening purpose and chronic THC abusers having a high concentration of drugs [88].

**Fig. 4:**
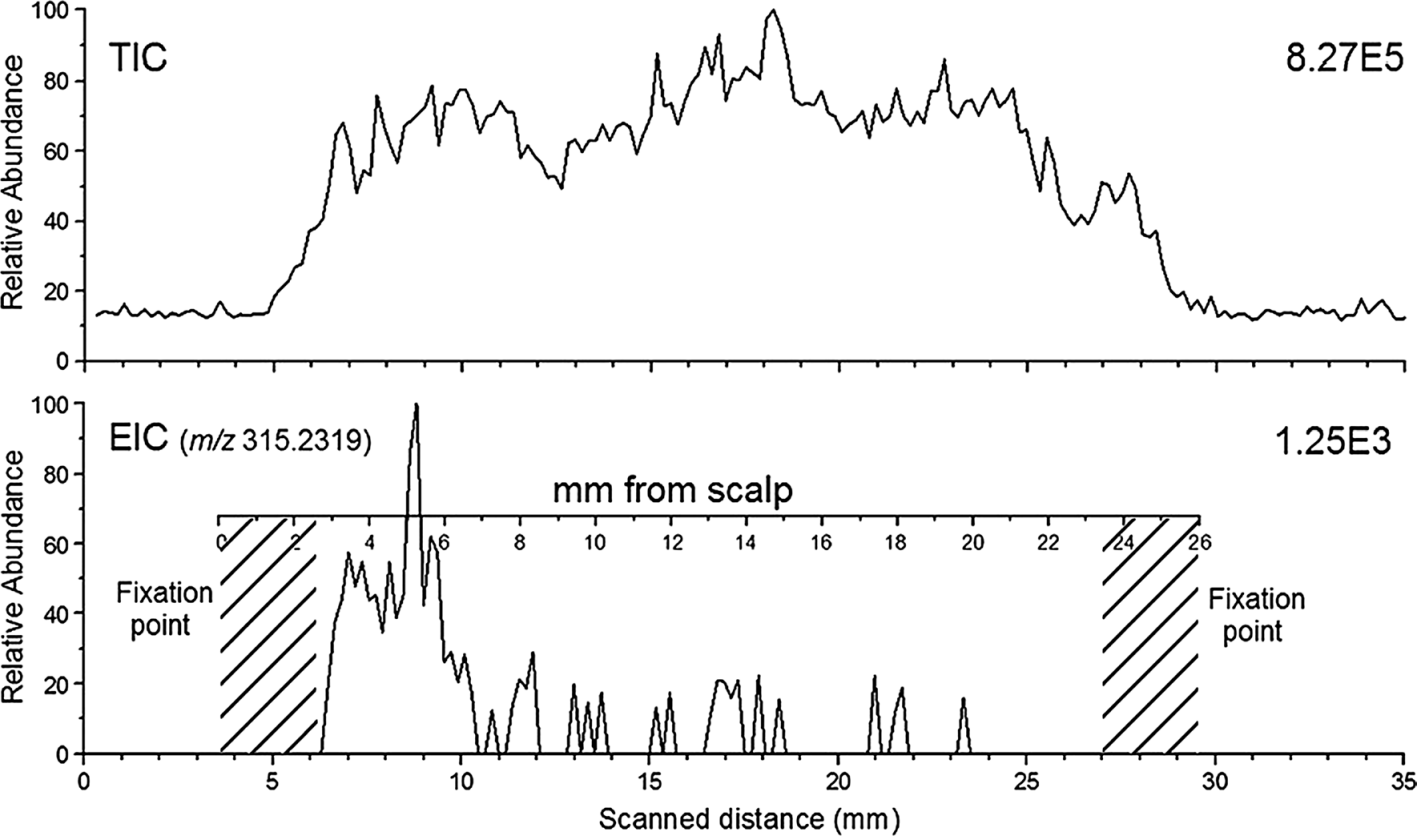
TIC and EIC of *m/z* 315.2319 from a DART hair scan (scan rate = 0.2 mm/s) of a cannabis user hair sample (reproduced with permission from John Wiley and Sons, reference, [88])

Figure 4 shows the TIC and EIC of m/z 315.2319 from a DART hair scan of a specific individual cannabis user hair sample. The resulting hair scan found the following results depending on the length from root to the tip, the first 2.5 to 7 mm, in which a lot of THC was detected, a second section, from 7 to 14.5 mm, where very small amount of THC was detected, and a third and last section, from 14.5 to 23.5 mm, where almost no THC was detected. The above sections were related to 1.5, 2.5, and 3 weeks of growth history of the hair sample. The results indicated that the scalp end of the sample contained more THC as compared to the tip end of the hair. The result of Fig. 4 shows that the DART hair scan can distinguish changing THC levels in different longitudinal sections of a hair sample and would have potential applications in the long term past retrospective timeline assessment of incidental drug intake [88].

## Cannabis and its metabolites

Cannabis and its metabolites are widely used as a drug of abuse due to its anxiolytic and euphorigenic properties. The determination of cannabis in the human specimen is very crucial for different phenomena such as therapeutic drug monitoring, doping control, driving licenses renewal, and drug-related deaths. In recent years, hair specimen has become a very crucial biological evidence and can be used alternative to conventional samples like urine and blood [11, 12]. However, hair is a very complex matrix, and it possesses a very small amount of drug in the range of Femto- to the pictogram, and it requires an additional extraction step and very sensitive analytical techniques for quantitation [97]. To achieve this level of sensitivity, there are various steps used for hair specimen analysis like decontamination, extraction, purification (cleanup) and finally detection and quantification in sub-picogram level.

### Detection

Cannabis could be identified in the human sample by varieties of techniques such as chromatographic techniques (LC–MS and GC–MS), Immunoassays, and Electro-kinetic methods [89, 123]. Immunoassays (IA) techniques can be used as screening methods that give a quick result that is later confirmed by chromatographic techniques like (LC–MS and GC–MS) [55, 90]. GC–MS is regarded as a gold standard, and it is used as a confirmatory technique for the detection of drug of abuse specifically for cannabis metabolite in hair [70, 93]. GC–MS needs derivatization of desire analytes before instrumental analysis, which lengthens the process. GC–MS is inexpensive and sensitive then LC–MS and more commonly used for synthetic cannabinoids having a various polar functional group that could be easily derivatized and detected [65, 78, 85, 86]. LC–MS is a Gold standard, analytical techniques, most widely used in toxicology labs for the detection of drug of abuse [90]. LC–MS has more sensitivity, and it is more rapid as it does not need a derivatization steps but it is more susceptible to matrix effects that consequently effect Linearity, accuracy, LOD and LOQ parameters during method validation [83]. Chromatographic techniques need a huge amount of hair sample (10–50 mg) and segmental analysis will make it more laborious for scientist. More recent advance techniques like MALDI-MS imaging and DART-MS are more easy to use techniques with rapid pre-screening capabilities for THC determination in hair [88, 115].

### External contamination

Although the usages of hair sample for detection of drug of abuse is highly valuable sometime, it gives us a false-positive result because of environmental contamination of drug incorporation into hair matrix [16, 36, 37]. Secondly, THC and its compounds are poorly bound to melanin that results in very low incorporation of drugs into the hair matrix [47]. The Sachs and Uhl diagnosed THC in two hair specimens where one partner was an active marijuana user, and the other one refused to take drugs and give false-positive result [70]. To overcome a false-positive result, the scientists used another useful marker like THC-COOH and Δ9-Tetrahydrocannabinolic acid A (THCA-A) that can provide details of external contamination [47, 83, 93]. The process of decontamination of hair with methanol, dichloromethane, or 5 g/L dodecyl sulfate in water can also reduce the chances of false-positive result [36]. To confirm the process of decontamination the treated and untreated hair sample in the exposure chamber will be processed in GC–MS, and their result compared [36]. External contaminants can be removed entirely with high efficacy using methanol and dichloromethane, followed by washing with dodecyl sulfate in water. Literature search has also shown that exposures of hair to marijuana smoke also cause contamination, which also depends on hair care habits and cosmetic treatments [51].

## Concluding remarks

This review presents the ‘state of the art’ in THC hair analysis for the past 10 years, including exploration and critical evaluation of the challenges and problems with the analysis of THC metabolites. For cannabis metabolites, the usual analysis that is performed involves the use of saliva, urine, and blood tests, whereas hair analysis has mostly been minimally explored. Mostly urine is the primary matrix that is used for monitoring drugs of abuse for forensic applications and drug abuse prevention. Saliva, urine, and blood require extensive handling, but they are prone to contamination and fiddling, and it has a shorter window of detection whereas hair analysis has a wider window of detection up to a year. Hair analysis is mostly used as a complementary test for urine, blood, and saliva analysis as THC takes about 2 weeks to reach the hair shaft. As the concentration of THC is hair is very low, and it requires very sensitive instrumentation while immunoassays can only screen THC in hair, but for quantitative purposes, LC–MS/MS and GC–MS instrumentations are widely used. There has been increased consumption of THC in the public domain, and most evidence does not support the adverse effects of cannabis contamination currently. Systematic scientific testing of cannabis is needed to monitor current and ongoing trends in cannabis potency, and to determine whether cannabis is contaminated. Additionally, more research is needed to determine whether increased potency and contamination correctly translate the harm it causes the users, and these users need to be provided with accurate and credible information on how to prevent and reduce harms associated with cannabis use. All of these considerations play an important part in increasing the enthusiasm and need for the analysis of hair for THC metabolites as a complementary test to urinalysis, saliva, and blood testing. For future work, it is imperative to set up the cut off limits for analysis of THC metabolites so it could be more beneficial in acting as a deterrent and abuse control.

## Data Availability

Please contact the author for data requests.
